# A Comparative Evaluation of Surface Properties of Cention N and TiO2-Enriched Cention N After Brushing Simulation and Erosive Challenge: An In Vitro Study

**DOI:** 10.7759/cureus.57048

**Published:** 2024-03-27

**Authors:** Nivetha Ambeth, Nancy Irudayaraj, Nikesh Sakthi, Deepika Lakshmaiah, Sadasiva Kadandale, Anupama Ramachandran

**Affiliations:** 1 Department of Conservative Dentistry and Endodontics, Chettinad Dental College and Research Institute, Chennai, IND

**Keywords:** erosion, roughness, nanoparticles, hardness, cention n

## Abstract

Background

This study aimed to evaluate and compare the abrasive and erosive wear resistance of Cention N and titanium dioxide (TiO_2_) nanoparticle-enriched Cention N after three years of brushing simulation.

Methodology

A total of 48 freshly extracted mandibular molars were mounted in acrylic blocks and divided into two groups of 24 molars based on the type of restorative material used to restore them. Cavities of a standardized size were prepared. Group A was restored with Cention N, and group B was restored with 5% TiO_2_-enriched Cention N. Each group was further divided into three subgroups of eight. Subgroup 1 was the control subgroup. Subgroup 2 was the abrasive subgroup, subjected to the abrasive challenge in a brushing stimulator with 30,000 cycles to 10,000 cycles in the linear X-axis and Y-axis each and another 10,000 cycles divided into 5,000 cycles clockwise and 5,000 cycles anticlockwise. The total number of brushing cycles was equal to three years of brushing with a duration of eight to nine hours. Subgroup 3 was the erosive and abrasive subgroup, subjected to an erosive pH cycle consisting of exposure to Coca‑Cola for five minutes thrice a day for seven days, and then subjected to brushing simulation as above. After the surface treatment, specimens were subjected to the Vickers microhardness test using a diamond indenter and the surface roughness test using an optical profilometer. The resulting values were subjected to statistical analysis.

Results

There was a significant decrease in mean surface roughness in group B, where TiO_2_ nanoparticles were added after erosive challenge and brushing simulation, than in group A. There was an increase in mean microhardness in group B which was not significant.

Conclusions

With the addition of 5% TiO_2_ to Cention N, there was a significant reduction in surface roughness. The surface microhardness of Cention N containing 5% TiO_2_ increased non-significantly compared to the control group.

## Introduction

The most common cause of tooth structure loss is dental caries, which impairs the shape and function of the affected tooth. Replacing the damaged tooth structure with proper restorative material is crucial to re-establish the esthetic, biological, and functional properties of tooth structure.

In dentistry, numerous direct filling materials are available, such as amalgam, glass ionomer cement (GIC), and composite material, with each having its own advantages and disadvantages [1,2].

With the characteristics of both GIC and amalgam, the new alkasite restorative material Cention N has added advantages. As a composite subgroup, Cention N has an alkaline filler, which can release ions that neutralize acid. It can release fluoride, calcium, and hydroxide ions. It can be used as a bulk fill material or can be light cured with blue light. It is aesthetically superior to amalgam and more translucent than GIC. It has higher flexural strength than GIC and amalgam and higher compressive strength than GIC. Lower polymeric shrinkage is seen in Cention N, which is translated into lower volumetric shrinkage, resulting in better marginal integrity.

Nanodentistry is an up-and-coming field in dentistry, and nanostructured materials are used in all aspects of improving dental health, ranging from diagnosis to treatment [3]. Nanostructured materials exhibit enhanced properties when compared to their bulk form. In biomedical research, nanoparticles of titania, silica, and alumina have been utilized successfully for enhanced mechanical strength and wear resistance [4-8].

Tests have been done with metal oxide nanoparticles with varying compositions, structures, shapes, and chemical and physical properties. From this aspect, titanium has been a major choice owing to its low toxicity, biocompatibility, chemical stability, and highly perceptible properties when compared to other metal oxide nanoparticles that are used for commercial purposes and are given more importance for research.

The aim of this in vitro study is to compare and evaluate the surface roughness changes and surface microhardness of Cention N and titanium dioxide (TiO_2_) nanoparticle-enriched Cention N after erosive challenge and toothbrushing simulation.

## Materials and methods

A total of 48 human molars extracted for periodontal reasons were selected and cleaned with ultrasonic scalers. Until further use, the teeth were stored in 0.1% thymol. The teeth were mounted in acrylic resin blocks using Teflon mold and divided into two groups based on the restorative material used to restore them. A standardized class 1 cavity measuring 6 mm × 4 mm × 1.5 mm was prepared on the occlusal surface of each tooth.

Experimental groups

A total of 48 samples were randomly divided into the following two groups of 24 samples each, based on the restorative material used: (1) Group A: Cention N (Ivoclar-Vivadent, Mumbai, India; n = 24) and (2) Group B: 5% TiO_2_ nanoparticles (Techinstro) enriched Cention N; n = 24).

Preparation of titanium-enriched Cention N

Cention N powder was blended with TiO_2_ nanopowder, anatase phase, 5% (w/w). The average particle size of TiO_2_ nanoparticles was 30-80 nm and the shape was near-spherical. Cention N powder and TiO_2_ nanoparticles were mixed for one minute in a vortex.

Placement of restorative materials

Group A was restored with Cention N, and, for manipulation, one scoop of powder was taken for one drop of liquid. Similarly, group B was restored with 5% TiO_2_-enriched Cention N. Each group had the following three subgroups with eight samples in each subgroup: (1) Subgroup A: control subgroup (n = 8), subgroup B: abrasive subgroup (n = 8), and subgroup C: erosive and abrasive subgroup (n = 8).

The samples of the control subgroup were placed in a container of artificial saliva throughout the experimental period.

**Figure 1:**
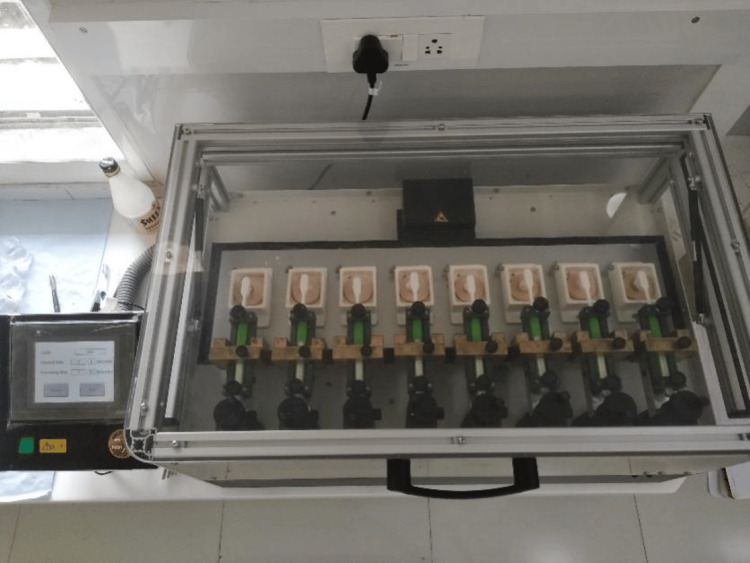
Brushing simulator.

For the abrasive subgroup, the samples were subjected to a toothbrushing simulation. In the brushing simulator (ZM3.8 SD Mechatronik), the samples underwent eight hours of brushing. The total number of cycles was 30,000, of which 10,000 cycles were performed in the linear X axis, and, in the linear Y axis, 10,000 cycles were performed. The final 10,000 cycles were further split into 5,000 cycles in a clockwise direction and another 5,000 cycles in an anticlockwise direction. Hence, the 30,000 cycles were equal to three years of brushing and were performed to evaluate long-term variations in the surface properties of Cention N and TiO_2_-enriched Cention N restorative materials. For the erosive and abrasive subgroup, the samples were stored in containers of Coca-Cola for a total of 72 hours. This testing period protocol was adopted from von Fraunhofer and Roger’s study for enamel dissolution in beverage solutions. Subsequently, the samples were subjected to the brushing simulation (Figure 1).

Surface microhardness evaluation

**Figure 2:**
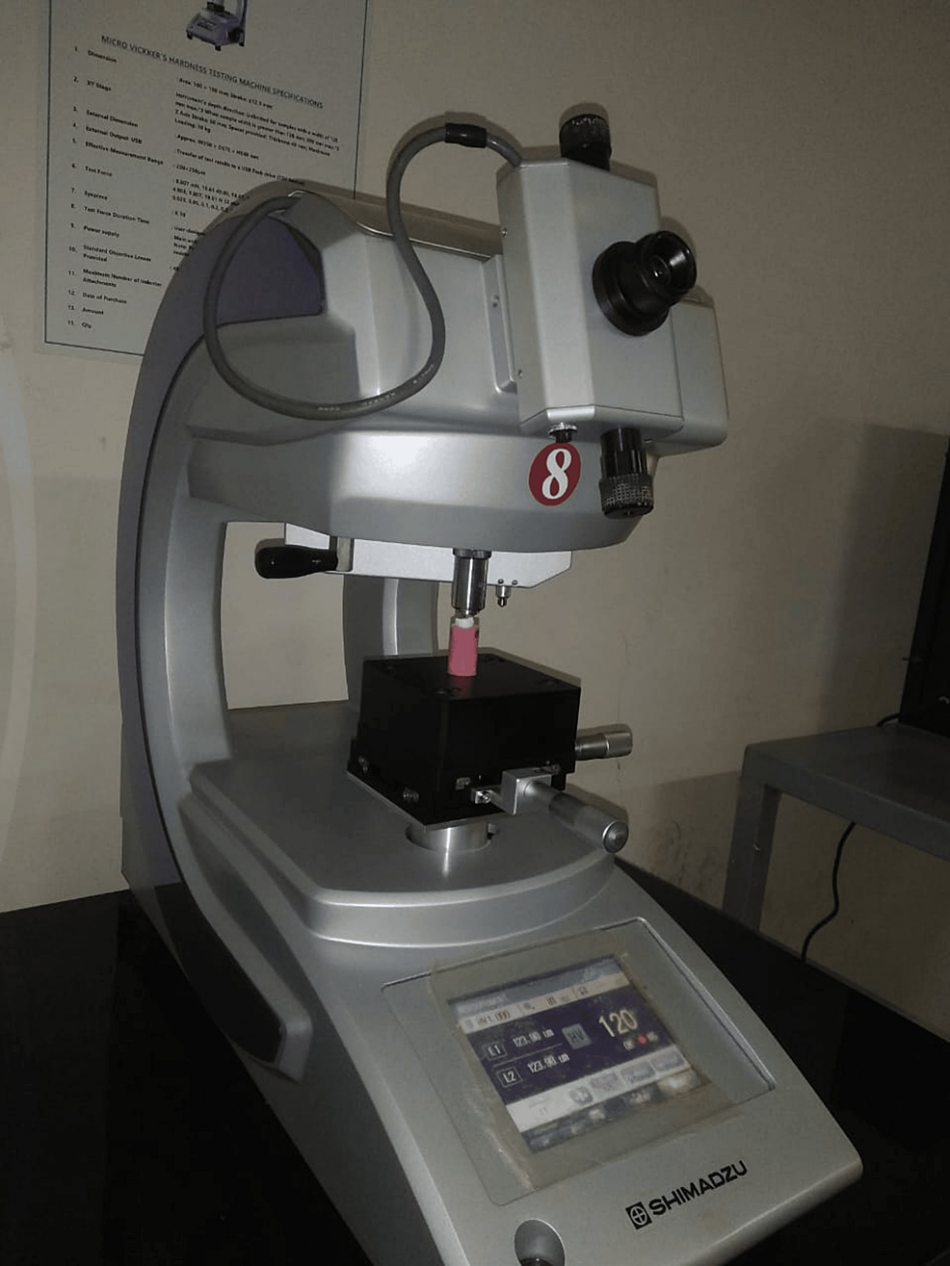
Vickers microhardness tester.

For surface microhardness analysis, a micro Vickers hardness testing machine with a diamond intender was used, and a load of 9.8 mN was applied for 10 seconds. Three indentations per sample were taken, and the average was calculated (Figure 2).

Surface roughness analysis

**Figure 3:**
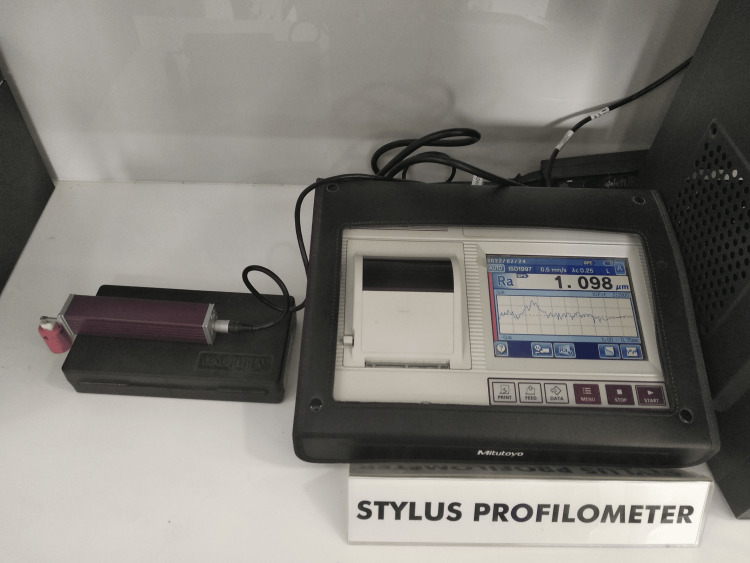
Stylus profilometer.

The surface roughness (Ra) was measured using a stylus profilometer (Figure 3).

Statistical analysis

The readings were tabulated and subjected to statistical analysis using a one-way analysis of variance (ANOVA) test. Mean and standard deviation were calculated for each group using the ANOVA test. P-values equal to or less than 0.05 were considered statistically significant.

## Results

The mean surface hardness value for each group is presented in Table 1. There was a significant difference in the surface roughness between group A and group B (p < 0.05). Hence, in the analysis of surface roughness, TiO_2_-enriched Cention N had lesser surface roughness than the Cention N group. As shown in Table 2, there was no significant difference in mean microhardness between groups A and B.

**Table 1:** Intergroup comparison of mean values of surface roughness (Ra).

Groups	Mean	SD	95% CI for mean		P-value
			Lower	Upper	
Cention N – Control group	0.7512	0.09949	0.6681	0.8344	0.001
Cention N – Abrasion group	1.6081	0.19320	1.4466	1.7696	
Cention N – Erosion and abrasion group	1.6512	0.16454	1.5137	1.7888	
Cention N + TiO_2_ – Control group	0.3156	0.19980	0.1486	0.4827	
Cention N + TiO_2_ – Abrasion group	0.4491	0.21592	0.2686	0.6296	
Cention N + TiO_2_ – Erosion and abrasion group	1.1240	0.23973	0.9236	1.3244	

**Table 2:** Intergroup comparison of mean values of Vickers microhardness.

Groups	Mean	SD	95% CI for mean		P-value
			Lower	Upper	
Cention N – Control group	139.1250	11.43225	129.5674	148.6826	0.236
Cention N – Abrasion group	137.0375	32.90262	109.5302	164.5448	
Cention N – Erosion and abrasion group	120.9625	16.88904	106.8429	135.0821	
Cention N + TiO_2_ – Control group	143.0000	15.06178	130.4080	155.5920	
Cention N + TiO_2_ – Abrasion group	143.0000	6.11789	137.8853	148.1147	
Cention N + TiO_2_ – Erosion and abrasion group	135.5000	22.19395	116.9454	154.0546	

## Discussion

Long-term durability is the basic requisite of every restorative material [9]. Many factors are involved in determining the surface properties of restorative materials, where daily oral hygiene measures play an important role. While toothbrushing aids the removal of plaque, its use along with toothpaste helps increase plaque control [10]. Toothbrushing influences the surface roughness of restorative materials, where the resulting abrasion could increase the accumulation of dental plaque [11]. Apart from this constant, pH cycles prevail in the oral environment, which could modify restoration properties and decrease durability [12]. In addition, consuming soft drinks with low pH was found to initiate erosive wear in restorative materials [13]. Coca-Cola was used in this study, as it is a popular soft drink with a very low pH. It has phosphoric acid and carbonic acid which favor dissolution and erode restorative materials easily [14]. For standardizing, abrasion specimens were extraorally brushed in a toothbrushing simulator, where the samples underwent eight hours of brushing. The total cycles were 30,000, of which, in the linear X axis, 10,000 cycles were performed, and, in the linear Y axis, 10,000 cycles were performed. The final 10,000 cycles were further split into 5,000 cycles in a clockwise direction and 5,000 cycles in an anticlockwise direction. Hence, the 30,000 cycles were equal to three years of brushing and were performed to evaluate the long-term variations in the surface properties of Cention N and TiO_2_-enriched Cention N restorative materials. In erosive subgroups, the specimens were stored in a container of Coca-Cola for 72 hours, which is equal to three years of drinking the soda, according to von Fraungafer and Rogers [15].

TiO_2_ nanoparticles were added to Cention N, as it has numerous promising properties, such as biocompatibility, non-toxicity, and chemical stability [16]. Hence, they have been suggested to be used in epoxy and resin composites as reinforcing fillers. Some studies reported that there is an increase in the strength of GICs by incorporating TiO_2_ nanoparticles. Moreover, these nanoparticles have been proposed as reinforcing fillers in dental resin composites and epoxies. In earlier studies, TiO_2_ nanoparticles have been utilized to improve the antibacterial properties and bond strength of composites. The antimicrobial effect of TiO_2_ nanoparticles against bacteria and fungi has been demonstrated by Lopez et al. (2012). Haghi et al. evaluated the antibacterial effect of TiO_2_ nanoparticles on *Escherichia coli* and showed that TiO_2_ nanoparticles create tiny pores in the cell walls of bacteria, leading to increased permeability and cell death [17]. Furthermore, they are used to improve the setting time, working time, compressive strength, and pushout bond strength of cement. Moreover, dental polymers were incorporated with silver-doped titania nanoparticles to produce bactericidal effects.

The surface roughness of both groups was tested in the control group, abrasive group, and erosive and abrasive group [18]. Our study showed a significant decrease in the surface roughness of TiO_2_-enriched Cention N.

In group A, where Cention N was tested, there was an increase in surface roughness after the toothbrushing simulation and after the erosive cycle, which was followed by the abrasive cycle. However, the increase was not significant, and their mean value indicated that erosion had no greater effects on the surface roughness of Cention N.

TiO_2_-enriched Cention N resulted in significantly less surface roughness even after the abrasive and erosive challenges. Although the exact mechanism is not known, it is possible that TiO_2_ improved the material’s homogeneity and consistency, decreasing the air void formation and microcracks in the cement matrix [19]. The TiO_2_ nanoparticles incorporated into the Cention restorative material filled the spaces between the particles of the Cention restorative material, which, in turn, enhanced the homogeneity and consistency of the modified version of TiO_2_ cention restorative material, resulting in increased microhardness.

A smooth surface is crucial because a rough surface may pave the way for plaque deposits, restoration color change, microleakage, and secondary caries [20]. Bacterial plaque retention is closely related to surface roughness, and one study showed that rougher composite surfaces exerted stronger bacterial adhesion [21]. Hence, the addition of TiO_2_ nanoparticles was shown to be promising in reducing the surface roughness.

The microhardness test measures the resistance of a material’s surface to plastic deformation through penetration [22]. This study showed an increase in microhardness in the TiO_2_-enriched group, but the increase was not significant. Abrasion and erosion did not affect it. This is similar to a study where Al_2_O_2_ nanoparticles were added, showing a significant increase in microhardness. The result could be attributed to the small size of TiO_2_ nanoparticles. The empty spaces among the larger glass filler particles of Cention N could be occupied by nanoparticles and offer the organic monomer part additional binding sites. It is in accordance with the study by Aref and Abdallah where there was an insignificant increase in microhardness by the addition of nanotitania to Cention N [23].

The addition of 5% TiO_2_ nanoparticles caused a reduction in surface roughness compared to the control group, while it increased surface microhardness. Hence, it can be concluded that incorporating TiO_2_ nanoparticles enhanced the surface properties. Thus, TiO_2_ nanoparticles along with their antibacterial properties can improve surface properties.

A significant limitation could be the in vitro design as the study was conducted in an environment that did not fully reflect the clinical situation. Hence, long-term clinical studies are necessary to support our conclusions. Moreover, three-year equivalent toothbrushing simulations may not fully capture the dynamic and multifaceted nature of long-term clinical scenarios.

## Conclusions

This study investigated the effects of the addition of TiO_2_ nanoparticles on surface roughness and surface microhardness of Cention N under different abrasive and erosive challenges.

Within the study’s limitations, we found that adding 5% TiO_2_ nanoparticles to Cention N resulted in a significant reduction in surface roughness compared to unmodified Cention N. The addition of 5% TiO_2_ nanoparticles improved the surface microhardness of the tested materials, although statistically insignificant, compared to the unmodified Cention N. As it is still not clear how the physicochemical mechanisms between nanoparticles and cement are realized, further studies must be conducted.
